# The Gas School of the American Expeditionary Forces

**Published:** 1918-12

**Authors:** 


### THE GAS SCHOOL OF THE AMERICAN EXPEDITIONARY FORCES

In delivering an address to the American medical officers recently assembled in Paris for a course of instruction in " Gas Poisoning ", Professor Roger, Dean of the Faculty of Medicine of the University of Paris, in addition to his cordial words of welcome, voiced the sentiment of the Allies regarding the deadliest and most inhuman method of warfare that has yet been devised, when he said : " Human science has introduced into the world evils without number, but the most horrible of all is the invention of poisonous and asphyxiating gases for purposes of warfare.

" In previous times, men fought openly and honorably, face to face; today men are poisoned to death by the products of the chemical industry released from a distance. Thus Germany has turned to evil, and to the destruction of mankind, those very forces which should have been enlisted on the side of progress and humanity. We are assembled here as allies in science as well as in war, in order to study how to encounter and overcome the evil effects of this German perversion of science. In order to crush the invader, our scientists will be co-operating with equal ardor to overcome the cruel effects of our enemy's barbarous and disgraceful methods of warfare. "

Those who had the good fortune to attend this school were very enthusiastic over the addresses and practical demonstrations which were given. As in all other endeavors on the part of the Medical Department of the American Expeditionary Forces to give its officers information necessary for the efficient performance of their duties in caring for American soldiers, our French and British medical confreres gave their cordial assistance in conducting this school. A number of addresses were given by eminent French, British and American physicians, who have made special investigations and important contributions on the various chemical and clinical phases of the gas problem.

Colonel Gilchrist, the efficient head of the Chemical Warfare Service of the American Expeditionary Forces, arranged this course of instruction. It should also be said that he and his associates, before the cessation of hostilities, made wonderful improvements in methods for the prevention and treatment of gas poisoning.

Clinical investigations of gas poisoning have also been carried out by emiment American clinicians. The results of these investigations, as well as the advances made by the Division of Chemical Warfare Service, cannot be published at this time, but the Medical Department of the American Expeditionary Forces surely did its part to protect our soldiers from injury by the use of this inhuman instrument of warfare.

One of the best treatises on the symptoms and treatment of gas poisoning, that has yet appeared, is that prepared by Colonel Gilchrist, and sent out as a circular from the Chief Surgeon's Office. Colonel Gilchrist gives full credit to British and French sources for much of the information contained in this article. For the benefit of those who may not have received a copy of this important circular, and in order to provide all officers of the American Expeditionary Forces with a permanent copy, it is reproduced in this number of *War Medicine* as a Circular from the Chief Surgeon's Office.

